# Supplementary material for: Finding landmarks - An investigation of viewing behavior during spatial navigation in VR using a graph-theoretical analysis approach
Source: PLoS Comput Biol. 2022 Jun 6;18(6):e1009485. doi: 10.1371/journal.pcbi.1009485 (PMC9203010; doi:10.1371/journal.pcbi.1009485)
Supplement: Attachment — Submitted filename: Response_to_reviewers.pdf [file pcbi.1009485.s001.pdf]

Point-to-point Reply by the Authors to the Review Comments

Reviewer's Responses to Questions and the corresponding Author's Replies

Review on Walter et al. "Finding landmarks—An investigation of viewing behavior during spatial navigation in VR using a graph-theoretical analysis approach"

Reply by the authors to the comments by reviewer #1:

Over evaluation

The manuscript by Walter et al. exploits graph-theoretical methods for a quantitative analysis of viewing behavior during a complex spatial navigation in a VR environment. The approach is very innovative and may be looked upon as a pioneering study of quantitative research in navigation. Among other important new findings, results lead to a new data-based account of the concept of visual landmarks.

Thank you very much for this very positive evaluation.

Comments

1) The introduction is succinctly written and provides most of the background required to evaluate the authors' findings.

Thank you for this very positive evaluation.

2) Typo "occular" (l. 135) -- it occurs throughout the manuscript and should be corrected.

Done – Thank you very much. We corrected it, i.e., using ocular, throughout the revised manuscript.

3) The concept (l. 139) of the collider should be explain to the average reader.

Done – Thank you for raising this point. We added an explanation of this concept in the revised manuscript (page 4 and 5).

4) The section on defining gazes in VR is very informative and important. However, to evaluate the potential influence of data preprocessing and treatment of missing data on the results, it is often a strategy to run an analysis with different preprocessing parameters to demonstrate reliability of the findings. It is up to the authors to decide if such an analysis is useful.

> Done - Thank you for raising this point. In addition to the threshold for interpolation and the threshold for defining gaze clusters as applied in the manuscript (k=8, i.e., less than 8 samples of missing data and at least 8 samples on a collider), we calculated the graphs for the thresholds of k=7 and k=9 for all participants as an additional control. The mean over participants of the number of nodes in the gaze-graphs varies very little (~2) with a change of the parameter. The mean number of edges varies moderately (~20), although there is considerable variance and the resulting edges are not necessarily identical. However, the choice of the parameter does not appear to be critical and in case of doubt, k=7 implies smaller changes than a choice of k=9. This information is added in the result section with precise quantitative statements (page 8/9).

5) The graph partitioning is an important analysis for the current manuscript. I would like to encourage the authors to introduce the concepts separately using a few toy examples. This can be done in the Methods. In my opinion, the current manuscript can be read as a methodological tutorial. Adding more information here might increase the impact of the manuscript. The same applies to the hierarchy index as well as to the rich club coefficient. Please provide more introductory examples to explain the concept carefully.

> Done – Thank you for this helpful suggestion. We added additional paragraphs with detailed descriptions of the used graph measures and example figures of simple graphs in the method section of the revised manuscript ("Graph Theory – detailed explanation of used graph measures", page 28-32).

6) The reference "Schaeffer (2007)" is not included in the references.

> Done – Thank you for pointing out the mistake. We added the reference into the bibliography list.

7) Overall, the discussion is well-balanced, but should come with subheadings, which would make it easier for the reader to follow the thoughts of the authors.

> Done – Thank you for this suggestion. We improved on the sequence of paragraphs in the discussion and added subheadings in the revised manuscript.

8) While I agree that there are many new findings in the current work, the author should at least point to the temporal/sequential aspects of the navigation problem. For example, we look differently at the same scene when viewing it the second time. It might not be useful to include more analyses, however, potential aspects of the dynamics of navigation and how they relate to the current graph-based approach should be explained.

Done - This is a very important but also complicated aspect. In this publication, we chose specifically to use a simple graph (undirected and unweighted) to investigate what kind of information we can detect about the participant's principle behavior. However, we acknowledge that by considering additional aspects like for example familiarity, i.e. how often a house was viewed, can add further information to the graph and thus allow additional analyses investigating important aspects of spatial navigation especially when using directional and weighted graphs. Consequently, the applied analytical tools must cope with the additional complexity, which will be addressed in further studies. These aspects are shortly discussed in the revised paper (page 22/23).

Typos

- l. 103: "behavior"
- l. 157: "in all following analyses"
- l. 183: "We interpolate missing data"
- l. 235: delete comma before "a new house"
- l. 265: "The latter is a"
- l. 269: "sort by increasing size"
- l. 358: "To further"
- l. 404: "a 2-sigma threshold"
- l. 444: "average highest"
- l. 465: "reflects"
- l. 518: "systems is often"
- l. 552: "in 3D"
- l. 559: delete comma before "that" – done (throughout more occurrences) – to be done
- l. 601: "study are crucial"

Done - These typos are all corrected. Some occurred at additional places and are corrected as well.

Reply by the authors to the comments by reviewer #2:

Reviewer #2: This manuscript used free exploration of a virtual town to investigate how gaze behaviors can inform spatial navigation. The authors used a novel data analysis technique to categorize gaze behavior in 3D VR and they also applied various graph theory analyses to their eye tracking data. The main findings were that participants viewed the city as a whole entity rather than separate smaller entities, participants on average used 10 houses in the city as landmarks, and these 10 houses were highly interconnected with other houses that were not categorized as landmarks (i.e., formed a rich-club). Participants also spent more than half of their exploration time in view of 2 or more of the 10 landmark houses. The authors use their results to conclude that participants seek out landmarks and strategically choose landmarks that will allow them to navigate around most of their chosen environment. They also discuss the challenges in practicality and data analysis with ecologically valid mobile eye tracking studies. Finally, they suggest using further graph theoretical measures to expand upon their results.

The motivation for the study is compelling because using eye tracking in realistic VR situations is instrumental for discoveries that are ecologically valid. The methods used to categorize gaze were novel and can be very useful for future studies. The choice of using graph theoretical measures seems relevant for the task and seems like a good way to visualize very large quantities of eye tracking data. I think the results are very interesting, and help advance our understanding of how people learn landmarks during navigation, though I think the conclusions the authors draw do not quite reflect the data. I am left with some questions about the methods and results, but with some revisions, I believe this paper would be a valuable addition to the literature.

> Thank you very much for this very precise summary of our paper and the positive evaluation of it.

Major:
1. The main finding of the study is that participants on average chose 10 houses as landmarks and in the discussion, the authors connect that to participants seeking out landmarks and strategically choosing their placement. Another alternative explanation is that more distinctive landmarks were placed around the city among some non-distinctive landmarks. This criticism is potentially addressable by adding more information about the houses in the city. Was the number of buildings with unique properties placed evenly throughout the city?

> Done – Thank you for this important suggestion. The houses that were all individual in style, shape and size were randomly placed in the city to avoid typical city districts. We added a paragraph about this aspect in the method section “the virtual town of Seahaven” (page 25) and shortly discuss it in the revised manuscript (page 25/26).

Were there other distinctive features on nearby buildings that participants could have chosen as a landmark? Can this issue please be addressed in some form by the authors in terms of the features of landmarks that are within the rich-club vs. those that are not?

> Done - Point well taken. Building the city, we avoided typical landmarks like churches but houses varied also in size. To depict the features of the rich club houses and not rich club houses, we included a figure in the revised manuscript depicting all 10 gaze-graph-defined

landmark houses that are members of the rich club and 10 houses that are not part of the rich club (Fig. 10, page 26). We shortly discuss this issue in the method section of the revised manuscript (page 25/26).

2. The first issue hits on another issue related to the framing of the manuscript. The author bring up an antiquated and largely abandoned hierarchical model of spatial processing, the stage model from Siegel and White (Siegel and White 1975). It is not clear why this model is raised because the authors are not explicitly testing it (nor some of the other models mentioned in the introduction). The focus of the paper is on landmarks, an issue of practically universal consensus in terms of importance within models of spatial navigation, and not routes or survey knowledge. Thus, the intro should probably be reframed to reflect the focus on landmarks and not theoretical models of navigation, which are not directly tested here.

Done – Thank you very much for helping us to improve our story line. We agree with you and focus on landmarks instead of models of spatial navigation in the revised manuscript. We rewrote the introduction (page 3) and the discussion accordingly (pages 23/24).

In addition, the authors bring up “triangulation.” However, as mentioned in the first point, it is not clear whether subjects are simply looking at salient landmarks or actually looking at multiple (three or more) landmarks, which would be the requirement for something being “allocentric” (Ekstrom and Isham 2017). While the looking patterns are connected amongst landmarks, the analyses in the paper can’t really distinguish looking at three or more landmarks as part of “triangulation” or simply using a single landmark as a beacon or orienting cue. This issue should probably be clarified, unless the authors are able to perform additional analyses that can show conclusively 1) subjects are not simply fixating salient (compared to non-salient) landmarks 2) subjects are consistently fixating three or more landmarks when they are viewing “connected” landmarks within the rich-club, and then using this to orient, in some form.

Done – We apologize for the imprecise phrasing and corrected this in the revised manuscript. We give a short definition of the use of triangulation. Then we carefully differentiate between what our data show and what the participants might have done or not but we do not know (e.g., actually using these landmarks for triangulation). Further, we discuss these aspects in the light of the existing literature, including the article by Ekstrom and Isham (2017). These changes can be found in the result (page 18) and discussion sections (page 24).

Minor/Detailed concerns:

3. The authors don’t specify clearly if Seahaven is a real place or if it was a fictional place created for the purposes of this experiment. I suspect that it is not a real place based on the description but having that clarification would be nice.

Done – You are absolutely right. We added a sentence to our Method section clarifying that “the virtual town of Seahaven” is a fictional place and was only created for the purpose of the experiment (page 25).

4. What were the specific instructions given to the participants for the task? There is very little information about the actual exploration. As I understand it, participants were not given any specific tasks during the exploration period except to explore the entire environment. Is that correct?

> Done – At the beginning of the experiment, before the exploration phase, the participants were introduced to the spatial tasks (orientation of a building towards north, orientation between houses, pointing between houses) that they had to perform after the exploration. Otherwise, they were instructed to freely explore Seahaven. We added a more detailed description of the instructions and the tasks in the method section of the revised manuscript (page 26/27, section "Structure of the experiment").

5. I think the authors go too far with their assumption that participants seek out landmarks, especially in strategic positions. Without knowing more about the configuration of the houses and if the salient features on the houses were equally distributed, I cannot accept this conclusion. Seeking out landmarks also implies that participants are consciously choosing landmarks to orient themselves but I do not think the data can inform us about incidental vs. intentional learning in this task given that participants could freely explore the environment and did not have to memorize the positioning of houses, nor engage in any kind of knowledge test of their environment after.

> Done – Point well taken. We do not claim that participants strategically selected landmarks for the purpose of navigation and deleted all statements that could give such an impression. This said, we give more context on the experimental design (König et al, 2019 and 2021). That is, after the free exploration of the city, participants were indeed tested on three spatial tasks (judging the orientation of a house towards north, judging the orientation between houses, and judging the direction between houses using a pointing task). Participants were informed about theses tasks before the exploration. So, they knew what they were tested on beforehand and were free to attend relevant features but we did not additionally instruct them accordingly. We give all information of our study design including the spatial tasks in the paragraph "Structure of the experiment" in the method section (page 26/27) of the revised manuscript.

6. On line 163, the authors define a time scale of "266 ms which is equivalent to eight data samples". I am unsure how the authors calculated this specific value.

> Done - The value is chosen based on the available hit point cluster length closest to the length of typical fixation durations. We give a detailed argument in the result section on page 6 including a reference to relevant literature. In short (and colloquial form), based on the 30 Hz sampling rate, a hit point sample is created after every 33.33 ms. Therefore, the hit point clusters closest to a typical fixation duration of 250 ms have a length of 233.33 ms, which is equivalent to a cluster of 7 hit point samples, and 266.667 ms, which is equivalent to the length of 8 samples. We choose the more conservative threshold of 266.667 ms, thus a hit point cluster consisting of 8 samples. In general, the duration of fixations is much longer than the duration of saccades. When the length of the missing data segment is shorter than a typical fixation duration and the gaze location just before and after the gap is on the same collider, it is reasonable to assume that no saccade-fixation-saccade back sequence goes undetected and to interpolate the missing samples. When the length of a continuous stretch of samples on a collider is at least as long as a typical fixation duration it is reasonable to

assume that this cluster of samples contains at least one fixation. We have extended the explanation of the threshold calculation and selection on page 6 in the revised manuscript.

7. In Figure 2b, the authors combine “all cluster durations longer than 1000 ms” in the last bin. I am unsure how this enhances the visualization of the graph. What information does this last bin represent? And how far after 1000 ms are there existing samples?

Done – Thank you for this question. The longest duration of a hit point cluster on a house is 18.9 s. Since the right tail of the distribution is very long, the area of interest between 0 and 1000 ms was not very well visible in the original histogram. Instead of zooming into the histogram to visualize the details of the first 1000 ms, we decided to accumulate all of the remaining right tail of the distribution and visualize it in the last bin. We improved the description of the figure caption in the revised manuscript on page (7).

8. On lines 258-259, the authors mention connections that are maintained and connections that are cut. I am not very familiar with graph partitioning and a description of what each of these terms mean would help my understanding of the analysis.

Done – Thank you very much for this suggestion. We improved the explanation of maintained and cut edges in graph partitioning in the revised manuscript (page 9/10). We took especially care to better explain all graph measures in the revised manuscript in the result section and added a specific paragraph in the method section that explains the measures on simple example graphs and corresponding figures (“Graph Theory – detailed explanation of used graph measures”, page 28-32).

9. In the graph partitioning section, I do not understand why the second smallest eigenvalue is used for all the further analyses. I also do not know what is gained from splitting the eigenvalues into positive and negative numbers and what the interpretation is for these numbers. An explanation to clarify each of these would be helpful.

Addressed – Thank you for raising this issue, which is not easy to address. In the revised manuscript we took several measures to remedy the problem. First, we gave a better explanation what the objective of this part of the analysis is. Second, we shortly mention an intuitive, albeit computationally rather ineffective approach, to explain the analysis. Third, we comment on some of the analysis steps, e.g., the second smallest eigenvalue. Finally, we provide introductory literature as well as more explanations in the method section. We do hope that this approach strikes a fair balance between readability, precision of the description, and providing pointers for those who are interested in depth of this analysis method.

10. The font in Figure 5 b-e is very small and hard to read.

Done – Thank you for this hint. We adjusted the font size in the revised manuscript (page 12).

11. In Figure 5b, were the participants ordered by node degree? I didn’t see anything in the text

about the participants being ordered for this graph but the way the data steadily increase makes me suspect they are in order.

Done – You are perfectly correct in your assumption. We added the information in the text (page 13) and improved the description in the figure caption (page 12, Figure 5).

12. I am confused by Figure 8a. The authors describe that "a value above 1 indicates the existence of a rich club with the respective node degree". The figure shows that values of node degrees have a value above 1, which would imply that all node degrees are a rich club instead of just the 10 houses that are most highly connected.

Done – We completely revised the text presenting the reasoning and explanation of the rich club coefficient. Specifically, we better explain the construction of the control graphs used for baseline. Next, we make it explicit that it is an index calculated for a threshold of node degree centrality. That is, all nodes with a degree at or above a threshold value are included. Next, we point out that the index is a continuous measure. Finally, we explicate the increase in variance of the estimated index that is necessarily occurring with the smaller set of nodes. These changes can be found on pages 16 and 17.

13. The current data show the top 10 most connected houses averaged over all participants but I would be interested in seeing which houses are most connected for each participant, perhaps to investigate any individual differences.

Addressed – We do show the distribution of node degree over participants in Figure 5c. There, the node degrees of all houses are compared over participants and we determine the correlation coefficients. In the revised manuscript we explicitly emphasize this consistency over participants and improve on the description of Figure 5. Further, in the revised manuscript we supply images of these top 10 houses to allow an individual evaluation of their features in (Fig. 10, p. 26). We are hesitant to dive into a full-fledged individual participant analysis, as this would add considerable material to an already long paper and potentially defocus the main story.

14. The paragraphs in the discussion about using mobile eye tracking to study more ecologically valid situations does not seem to flow in the discussion. It sounds more like the motivation that fueled the study and perhaps should be integrated into the introduction.

Addressed – We agree with the evaluation of the problem. However, we were hesitant to extent the introduction noticeably. Therefore, we rewrote the introduction, mention the motivation shortly, and rearranged and revised the material in the discussion (pages 3/4, and 20/21). We hope that the flow of the discussion is improved and the motivation communicated early in this way.

15. The results showed that participants spent more than half of their time in the heart of the city instead of the outskirts, which the authors interpret as meaning that participants would rather be able to see 2 or more landmarks so they could triangulate their position. An alternative

interpretation is that participants were simply more interested in staying in the middle of the city because city centers tend to have a lot of interesting things to explore.

Done – This relates to issue #2 and a large part of the response applies here as well. Further, we refrain on any interpretation of why the participants spent their time in a specific area of the city. Additionally, in the revised manuscript we give more information on the design of the city in the method section (page 25/26). Specifically, we did not build Seahaven with a city center and suburbia areas. Instead, we aimed at distributing the houses that had to be unique and thus varied in style, shape and size randomly over the city area.

Very minor grammar concerns or typos:

1. Line 27: “allows to quantify” -> “allows one to quantify”
2. Line 66: “the own” ->“one’s own”
3. Line 180: “ensures to capture” -> “ensures that most fixations are captures while…”
4. Line 265: “latter a” -> “latter is a”
5. Line 267: “degree-“ had an extra –
6. Line 274: “visible in form” -> “visible in the form”
7. Line 358: “o” -> “To”
8. Lines 364-365: “For the example participant reported already above this results in a slope of -2.6349”. This sentence is confusing to me. Done – Sorry for our weak phrasing. We improved it in the revised manuscript (page 13).
9. Line 419: “allows to quantify” -> “allows one to quantify”
10. Lines 455 and 456: “infer the own” -> “infer one’s own”
11. Line 506: “a virtual reality” -> “a virtual reality task”
12. Line 510: “allows to perceive” -> “allows one to perceive”
13. Line 554: “allows to cleanly separate” -> “allows one to cleanly separate” ”
14. Line 602: “a few millions” -> “a few million” ”

Done - These typos are all corrected. Some occurred at additional places and are corrected as well.

In addition, we improved our data availability by archiving the Github repository containing all pre-processing and analysis script using Zenodo to add a persistent digital identifier (DOI) in addition to the weblink. We adjusted the data availability statement accordingly.
