# Supplementary material for: Finding landmarks - An investigation of viewing behavior during spatial navigation in VR using a graph-theoretical analysis approach
Source: PLoS Comput Biol. 2022 Jun 6;18(6):e1009485. doi: 10.1371/journal.pcbi.1009485 (PMC9203010; doi:10.1371/journal.pcbi.1009485)
Supplement: Attachment — Submitted filename: Response_to_reviewers_2Review.docx [file pcbi.1009485.s002.docx]

Reply by the Authors to the Review Comments

**Reviewer #1**: I would like to thank the authors for their revised version. Congrats!

Thank you very much.

**Reviewer #2**: #7. In Figure 2b, the authors combine “all cluster durations longer than 1000 ms” in the last bin. I am unsure how this enhances the visualization of the graph. What information does this last bin represent? And how far after 1000 ms are there existing samples?

Done – Thank you for helping us improve our figure. We changed the figure so it only displays the distribution of hit point clusters zoomed into the region of interest between 0 and 1033 ms without further modifications. Consequently, the last bin now displays the probability of hit point clusters of size 1033 ms. The longest hit point cluster on a house has a duration of 18.9 s (18900 ms). We have adjusted the figure caption accordingly.

**Regarding the data availability.**

We double checked all provided links. Furthermore, we updated the Zenodo archive DOI, since we made a small change to the repository. Now the new script that creates the toy examples we included in the methods section during the first review is also uploaded to the repository. Consequently, we needed to create an updated archive which results in a new Zenodo DOI. The change in the data availability statement is marked in the newly uploaded manuscript documents.

In general, all data is available at the center of open science and can be downloaded either by downloading each individual file or the whole folder as a Zip archive by following the instructions provided on the website.

All code is available at our Github repository. To access all the files provided in the repository, the repository can be either cloned or it is also possible to download the individual files or the whole repository itself.
